# Pre- and Postoperative Ultrasound and MRI Examinations in Assessing Vocal Folds in Patients with Goiter

**DOI:** 10.3390/diagnostics12061362

**Published:** 2022-06-01

**Authors:** Magdalena Derlatka-Kochel, Paweł Kumoniewski, Marcin Majos, Kamil Ludwisiak, Lech Pomorski, Agata Majos

**Affiliations:** 1 Department of Radiologic and Isotopic Diagnositcs and Therapy, Medical University of Lodz, 90-419 Lodz, Poland; magdalena.derlatka@umed.lodz.pl (M.D.-K.); k.ludwisiak@csk.umed.pl (K.L.); agata.majos@umed.lodz.pl (A.M.); 2 Department of General and Oncological Surgery, Medical University of Lodz, 90-419 Lodz, Poland; pawel.kumoniewski@umed.lodz.pl (P.K.); lech.pomorski@umed.lodz.pl (L.P.)

**Keywords:** MRI, ultrasonography, laryngoscopy, vocal fold paralysis, rima glottis, goiter

## Abstract

Aim of the study: To determine the value of dynamic examinations ultrasound (US) and MRI in the 1.5T field in the assessment of the mobility of vocal folds (VF) in comparison to laryngoscopy in patients with thyroid gland resection. Materials and methods: A total of 44 patients with goiter, before and after thyroidectomy, were subjected to videolaryngoscopy and dynamic examinations of the vocal folds using ultrasound and the following MRI sequences: generic gradient echo (GRE) and true fast imaging with steady-state precession (TRUFI). The qualitative and quantitative data were analyzed, i.e., the angles of deviation from the midline of the vocal folds and the area of the right and left rima glottidis compartments. Results: The analysis of qualitative data showed that the results obtained by laryngoscopy, US and MRI are independent of the diagnostic method used in the group of patients pre and post thyroidectomy. Between the pre- and postoperative examinations in the group of paralyzed vocal folds, statistically significant differences were found in the minimum and maximum values of the angles for the MRI-GRE and MRI-TRUFI sequences and the maximum value of the angles in the US examination, but also in the maximum value of the area of the glottis compartments in both MRI-GRE and MRI-TRUFI dynamic sequences and the minimum value of the area in the sequence MRI-GRE. Statistically significant differences were found in both MRI sequences during phonation, both for the value of the angles and the area of the affected vocal folds. However, no statistically significant differences were found in the values of the angles or the areas in both vocal fold imaging methods without identified mobility abnormalities. Conclusions: Ultrasound and MRI examinations using dynamic sequences have a similar diagnostic value to laryngoscopy in the assessment of vocal fold paralysis in patients with goiter. The GRE sequence seems to be the most reliable one in determining vocal fold paralysis, and the most reliable parameter is the maximum area of the rima glottidis compartment. The inclusion of dynamic short sequences widely available in 1.5T scanners in standard neck examination protocols represents a novelty of the method and a promising diagnostic perspective in the diagnosis of vocal fold paralysis.

## 1. Introduction

Pathologies of the thyroid gland which require a thyroidectomy affect a large part of the world population, mainly women [1,2]. One of the serious complications of this type of surgery is a paralysis of the recurrent laryngeal nerve which supplies the vocal fold [3,4,5]. The gold standard in the assessment of the vocal folds is laryngoscopy [6]. However, this method is poorly tolerated by patients, and most importantly, in the era of the COVID-19 pandemic, it is associated with a higher risk of infection [7,8].

Dynamic medical imaging methods such as ultrasonography (US) and magnetic resonance imaging (MRI) enable us to assess the function of anatomical structures, thus constituting potentially promising tools for the noninvasive assessment of mobility disorders of the vocal folds [9,10,11,12].

The objective of the study was to determine the value of dynamic examinations such as ultrasound and MRI using the GRE (generic gradient echo, Siemens, Erlangen, Germany) and TRUFI (true fast imaging with steady-state precession, Siemens, Erlangen, Germany) sequences in the assessment of the mobility of the vocal folds in patients with goiter before and after thyroidectomy compared to laryngoscopy.

## 2. Materials and Methods

The study was conducted from 2018 to 2020 after obtaining the approval of the Bioethics Committee at the Medical University of Lodz (decision no. RNN/187/18/KE of 15 May 2018).

The control group included 35 healthy volunteers (25 women, 10 men, aged 20 to 59; Me = 34). They had no hoarseness, chronic diseases of the thyroid gland or the upper respiratory tract or any prior head, neck or chest surgery.

The study group consisted of 44 patients from the Clinic of General and Oncological Surgery (39 women, 5 men, aged 18 to 70; Me = 43). Each patient underwent a wide range of tests: laryngoscopy, ultrasound and MRI. In 5 men, the ultrasound revealed massive calcifications in the thyroid cartilage, which made it impossible to visualize the vocal folds. Therefore, the group of patients undergoing ultrasound examination was smaller than the remaining groups, i.e., patients undergoing laryngoscopy and MRI tests. We entirely excluded from the study 5 women who did not consent to postoperative diagnostics due to increased pain in the operated area or significant discomfort during preoperative laryngoscopy.

**Figure 1:**
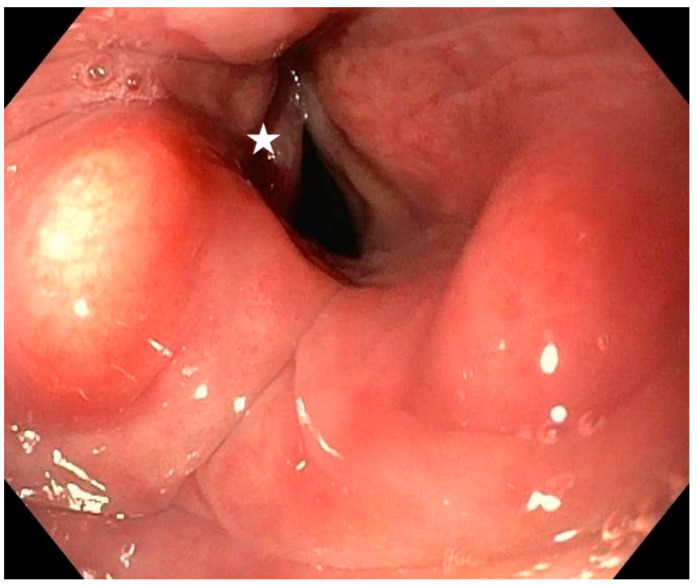
Asymmetry of the glottis during breathing on laryngoscopy. Left vocal fold paralysis (asterisk).

Laryngoscopy was the reference study to which the results of the dynamic radiological tests were compared. This examination was carried out in the Clinic of Surgery by the same doctor using a standard gastrofiberoscope via oral access (Figure 1).

**Figure 2:**
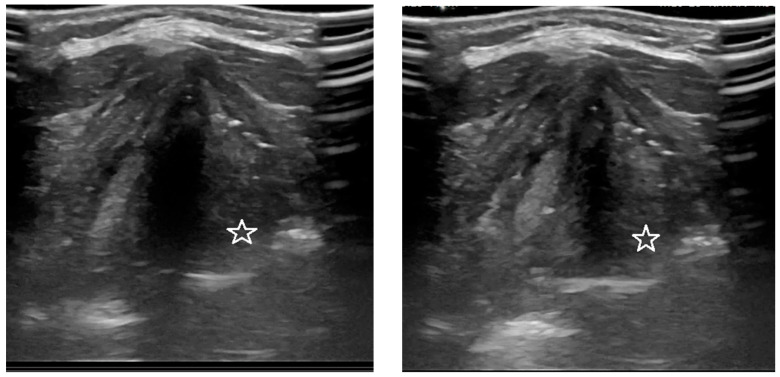
Asymmetry of the glottis during breathing on ultrasound. Reduced mobility and adduction of the left vocal fold (asterisk).

Ultrasound examinations were carried out with a Logiq S8 machine (GE Healthcare, Chicago, IL, USA) using an ML6-15 linear probe with a frequency range of 5–13 MHz. The following output parameters were used: frequency 8 MHz, depth of field of view 4–5 cm, gain (Gn) 43%, dynamic range (Dr) 69 dB. The obtained examination results consisted of images and videos. The ultrasound examination was conducted with the patient in a supine position with the head slightly tilted back. The probe was applied transversely to the long axis of the body, in the midline, at the level of the mid-segment of the thyroid cartilage. Moving the probe along the patient’s long axis, an image was sought that showed both vocal folds in the transverse plane. All of the above anatomical structures, the vocal folds, vestibular folds and arytenoid cartilages, were identified in each ultrasound examination. The mobility of the vocal folds was observed during normal breathing and phonation. The “heee” was used as the reference sound during phonation because the glottis is the narrowest at the time when the “e” vowel is pronounced. During the data acquisition, on the examiner’s command “start”, the subject produced the sound until they heard the word “stop”. Then, the presence of the respiratory dependent fold mobility was assessed visually—the maximum opening during inspiration and slight adduction on exhalation as well as the symmetry of the glottis (Figure 2). On the basis of the obtained material, the angles of the minimum and maximum deviation of the vocal folds from the midline were measured using the ImageJ program retrieved from https://imagej.nih.gov/ij/ (accessed on 1 July 2018).

**Figure 3:**
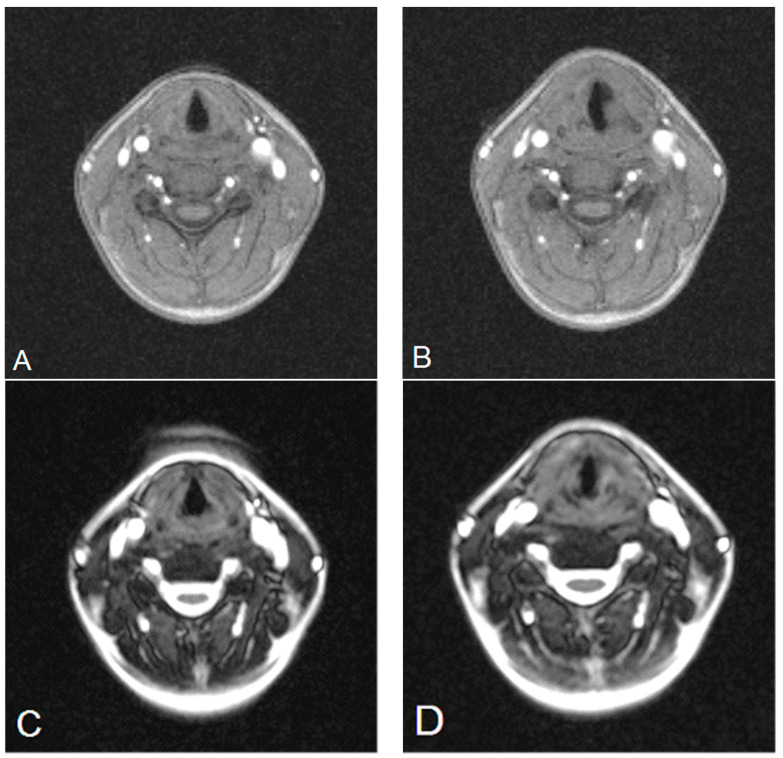
Images of left vocal fold paralysis (**B**,**D**) compared to preoperative examination (**A**,**C**). MRI-GRE (**A**,**B**), MRI-TRUFI (**C**,**D**).

MRI was performed with a 1.5T Magnetom Avanto scanner (Siemens, Erlangen, Germany) with the use of an eight-channel cervical coil and a four-channel head coil. No contrast agents were administered. For morphological evaluation, T2-weighted sequences were used: turbo spin echo in the transverse plane (TSE, Siemens; TR-3440 ms, TE-76 ms, IR-0.9 × 0.9, slice thickness-3, number of slices-25, flip angle-150, total acquisition time-4:42) and turbo inversion recovery magnitude in the frontal plane (TIRM, Siemens; TR-3600 ms, TE-82 ms, IR-0.8 × 0.8, slice thickness-4, number of slices-45, flip angle-127, total acquisition time-3:43). The following sequences were used for dynamic tests: generic gradient echo (GRE, Siemens; TR-23 ms, TE-3.64 ms, IR-1.1 × 1.1, slice thickness-6, number of slices-3, flip angle-20, total acquisition time-1:08, number of dynamic scans-15) and balanced gradient echo (TRUFI, Siemens; TR-364.76 ms, TE-1.29 ms, IR-1.4 × 1.1, slice thickness-10, number of slices-1, flip angle-62, total acquisition time-0:22, number of dynamic scans-15) in the axial plane. The assessment of the adduction and abduction of the vocal folds was performed with the use of the dynamic gradient sequences GRE and TRUFI during normal breathing (Figure 3) and phonation, also with the use of the “hee” sound. The slices were analyzed with the software provided by the scanner manufacturer (Syngo.via, Siemens, Erlangen, Germany). Each part of the rima glottidis compartment was measured using ROI Freehand function. The reference plane was the line passing through the anterior and posterior commissures. Both the glottal angle and the glottic area were measured between the aforesaid plane and each of the vocal folds, separately for the right and left compartment.

Statistical analysis of the results was carried out with Statistica 10 software (StatSoft, Tulsa, OK, USA). Qualitative variables (results of laryngoscopy, US and MRI) were verified using the chi-square test of independence. The analyzed data (vocal fold angles and surface area of the glottis) were quantitative. The Shapiro–Wilk test was used to determine the normality of the distribution. The distributions of the examined variables were not consistent with the normal distribution. Research hypotheses were verified using the Kruskal–Wallis, Wilcoxon and post hoc Dunn’s tests. The results were considered significant for *p*-values < 0.05.

## 3. Results

### 3.1. Analysis of the Qualitative Data Obtained during Ultrasound and MRI in Patients with Goiter Compared to Direct Laryngoscopy

The results obtained on the basis of laryngoscopy, US and MRI examinations of healthy volunteers and preoperative patients showed no abnormalities in the mobility of the vocal folds. Out of 44 patients after thyroid gland surgery, 9 women were diagnosed with abnormal movement of the vocal folds on laryngoscopic examination. One patient had bilateral vocal fold paralysis, whereas the remaining eight women had a unilateral limitation of vocal fold mobility. In total, this amounts to 10 vocal folds affected by the pathology. In this group of patients, dynamic ultrasound and MRI methods confirmed the presence of disorders.

The performed analysis of the qualitative data from the ultrasound, MRI and laryngoscopic examinations showed that in the group of patients before and after thyroid gland resection, the results obtained are independent of the employed diagnostic method (*p* > 0.05).

### 3.2. Analysis of the Quantitative Data Obtained Using Ultrasound and MRI Methods (GRE and TRUFI Sequences) before and after Thyroidectomy

**Figure 4:**
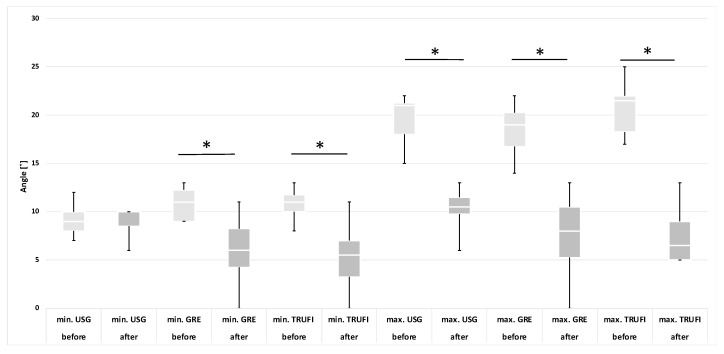
The minimum and maximum values of the glottic angles in the group of paralyzed vocal folds after surgery in relation to the preoperative examination with the use of US and MRI sequences (GRE and TRUFI). * *p* < 0.05.

**Figure 5:**
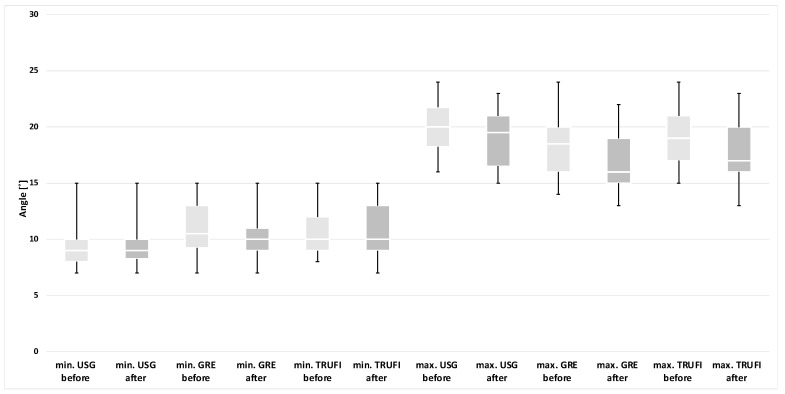
The minimum and maximum values of glottic angles in patients without changes in the mobility VF after surgery in relation to the preoperative examination with the use of US and MRI sequences (GRE and TRUFI).

**Figure 6:**
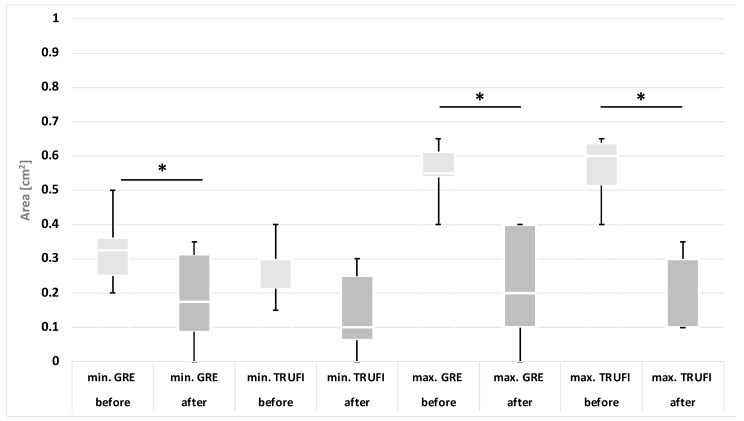
The minimum and maximum values of the area of the rima glottidis compartments obtained with the use of dynamic MRI sequences in the group of patients with paralyzed vocal folds after surgery compared to the preoperative examination results. * *p* < 0.05.

**Figure 7:**
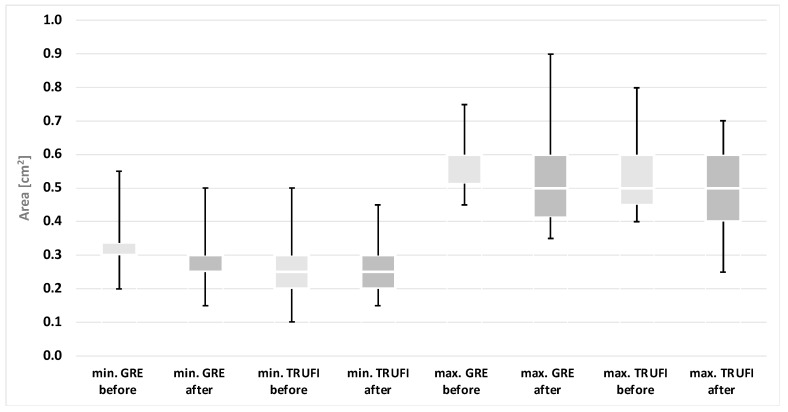
The minimum and maximum values of the area of the rima glottidis compartments obtained with the use of dynamic MRI sequences in the group of patients without VF mobility abnormalities in comparison to the preoperative examination.

Based on the results of the laryngoscopic examination performed after thyroid gland resection, patients were divided into two groups: with normal and impaired mobility of the vocal folds. A separate analysis was performed for each group, in which the minimum and maximum values of the angles and areas of each glottis compartment were compared with the corresponding values obtained during preoperative diagnostics. These results are presented in the form of graphs (Figure 4, Figure 5, Figure 6 and Figure 7). The measurements for both methods US and MRI in these two postoperative groups are presented in Table 1 and Table 2. Postoperative abnormal mobility of the vocal folds occurred only among women. Therefore, the results presented in Table 1 apply only to this gender. Table 2 presents the results of measurements for women in US and the whole group in MRI. It was caused by the inability to obtain measurements in men due to calcifications in the thyroid cartilage.

Glottic angle:

Among the paralyzed vocal folds, statistical differences were found between the minimum (*p* = 0.0059) and maximum (*p* = 0.0058) values of the VF angles for the MRI-GRE sequence, minimum (*p* = 0.0204) and maximum (*p* = 0.0137) values of the VF angles for the MRI-TRUFI sequence and the maximum value of the angles (*p* = 0.0057) obtained during US between pre- and postoperative examinations. No statistical differences were found in the minimum angles on ultrasound examination (*p* = 0.8902). The comparison between the values of the angles obtained during the preoperative and postoperative examinations is shown in the graphs (Figure 4). The ranges of the values of the minimum angles in pre- and postoperative examinations partially overlap in all diagnostic techniques, and the ranges of the values of the maximum angles are completely disjoint.

The group of people with no detected VF motion abnormality showed no statistical differences in the values of the angles on US and MRI (*p* > 0.05). A comparison of the values of the angles obtained for these patients is presented in the graph (Figure 5).

The area of the rima glottidis compartments:

In comparison with the preoperative tests, statistically significant differences were found in the maximum values of the glottis compartment areas for both the dynamic sequences MRI-GRE (*p* = 0.0049) and MRI-TRUFI (*p* = 0.0131) and the minimum values of the areas for the MRI-GRE sequence (*p* = 0.0138). However, no such differences were observed in the minimal areas for the MRI-TRUFI sequence (*p* = 0.0569). The comparison of the values obtained in the preoperative and postoperative tests is presented in the diagram (Figure 6). The ranges of the minimum values of the glottis area in the pre- and postoperative examinations for both MRI sequences partially overlap, and the ranges of the maximum values of the glottis area are almost disjoint.

In the group of people without vocal fold paralysis, no statistically significant differences were found in any of the area values obtained with the analyzed imaging methods (*p* > 0.05). The comparison of the values for these patients recorded during the pre- and postoperative examinations is presented in the diagram (Figure 7).

### 3.3. Analysis of the Quantitative Data Obtained with the Use of GRE and TRUFI Dynamic Sequences in the Group of Paralyzed Vocal Folds during Phonation

**Figure 8:**
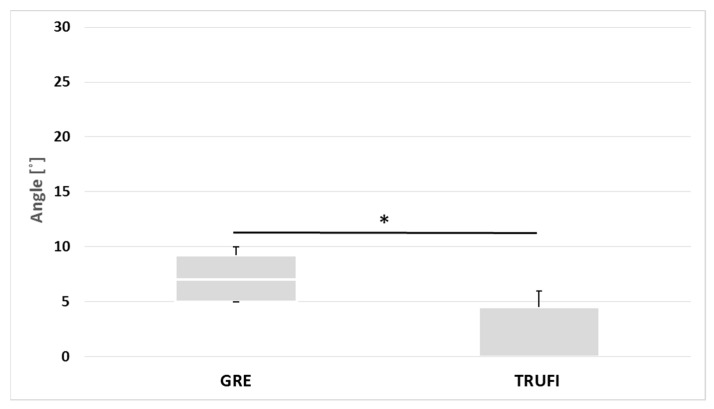
The values of the maximum glottic angles of the paralyzed vocal fold during phonation in MRI dynamic sequences. * *p* = 0.0136.

**Figure 9:**
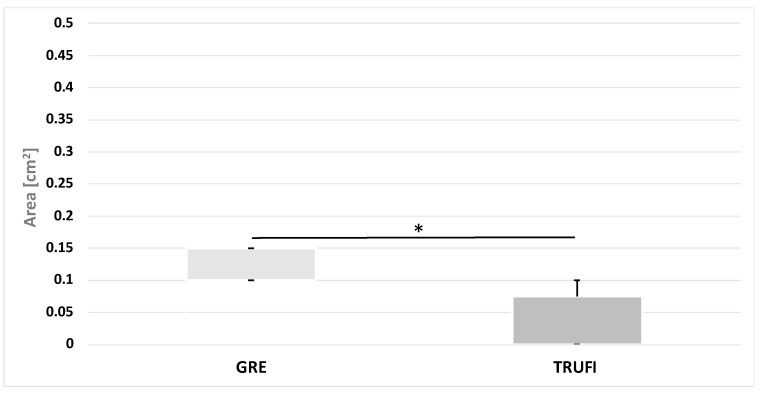
The values of the maximum area of the rima glottidis compartments on the side of the paralyzed vocal fold during phonation in MRI dynamic sequences. * *p* = 0.0119.

The values of the maximum angles and areas of the glottis on the side of the affected vocal folds were compared between the two sequences GRE and TRUFI during phonation. There were significant statistical differences between the two sequences, both in the value of the angles (*p* = 0.0136) and the area of glottis compartments (*p* = 0.0119) (Figure 8 and Figure 9). The ranges of the angle and area values obtained by the two sequences are almost disjoint.

## 4. Discussion

As a result of the dynamic technological progress in imaging diagnostics, it is possible to assess anatomical structures in motion and to conduct reliable and repeatable measurements. These criteria are met by two diagnostic methods, ultrasound and magnetic resonance imaging. US has the advantage of being widely available, cheap, easy to use and even at the patient’s bedside. The advantages of MRI are non-invasiveness, no exposure to ionizing radiation in comparison to computed tomography, the possibility of objective measurements and the assessment of the movement of anatomical structures with the use of short, fast dynamic sequences. These advantages formed the basis for determining the potential of both methods for vocal fold evaluation in patients with goiter.

In this study, we analyzed qualitative data based on ultrasound, MRI and laryngoscopy. The preoperative examinations using both US and 1.5T MRI imaging methods performed on 44 patients with goiter showed no abnormalities in vocal fold mobility during normal breathing. In the group of patients after resection of the thyroid gland, abnormalities in the mobility of the vocal folds were detected on laryngoscopic examination in nine women. One patient had complete and bilateral paralysis, while the remaining eight patients had unilateral paralysis. Out of all patients who were examined after surgery, complete paralysis accounted for 2.9% of the subjects, and partial paralysis for 23.5%. In each case of detection of abnormal vocal fold mobility during laryngoscopy, the pathology was also found on ultrasound and both MRI-GRE and MRI-TRUFI dynamic sequences. Statistical analysis showed that in the group of patients after thyroid gland resection, the results obtained on the basis of laryngoscopy, US and MRI are the same irrespective of the employed method.

These observations are consistent with the reports by other authors. In their latest prospective multicenter analysis and literature review, Gambardella et al. found that the results of vocal fold ultrasound and laryngoscopy were 95.7% consistent [13]. Shah et al. also confirmed that transcutaneous laryngeal ultrasonography is nearly as effective as videolaryngoscopy and fiberotic laryngoscopy in patients undergoing thyroidectomy [14]. They showed that US can serve as a non-invasive, bedside screening tool for assessing vocal cord palsy. The literature on the diagnosis of vocal fold paralysis based on dynamic MRI sequences is scarce. Schlamann et al. showed that dynamic MRI examination with the use of SSFP sequences belonging to the group of gradient echo sequences detects vocal fold paralysis to the same extent as laryngoscopy [15]. Only 12 patients were included in the study, and 7 of them were found to have abnormalities. Faust et al. using the dynamic turboFLASH sequence, which is also an echo gradient one, in a group of 10 children similarly showed no discrepancies between the MRI results and laryngoscopy in the diagnosis of VF paralysis [16].

Similarly, in evaluating the quantitative data, we did not observe any differences between healthy volunteers and preoperative goiter patients. The performed statistical analysis of the minimal and maximal values of angles of the vocal folds for the ultrasound method and both dynamic MRI sequences did not reveal significant differences: in the group of healthy volunteers, in the group of patients before the surgery and between the two groups, as well as in the group of patients after surgery without vocal fold paralysis and compared to their preoperative tests. In the group of patients before the operation, the standard deviation was relatively high for the value of the maximum angle in the TRUFI sequence compared to the other measurements. Although no statistical significance was found, these observations indicate that the values of the minimum and maximum angles may be affected by both data acquisition techniques and the type of sequence.

In the group of patients with vocal fold paralysis, the minimum and maximum angles of the vocal folds recorded with both imaging methods were significantly different between the pre- and postoperative examinations—except for one parameter, i.e., the minimum value of the angle determined on ultrasound examination. It proves that the diagnostic reliability of ultrasound is lower in comparison to both MRI sequences. Moreover, lower statistical significance characterized the GRE sequence compared to TRUFI for both the minimum and maximum angles. Therefore, it can be concluded that the GRE sequence is the imaging technique with the highest diagnostic value for the assessment of vocal fold mobility.

Similarly, based on the GRE and TRUFI dynamic sequences, no statistically significant differences were obtained for the minimum and maximum areas of the glottis compartments in the group of healthy volunteers, preoperative patients or between these two groups. Furthermore, no such differences for these parameters were found in patients after surgery without vocal fold paralysis or when comparing this group’s post- and preoperative test results. The values of medians and standard deviation for all analyzed groups were similar for both sequences. Based on the analyzed data obtained in the study, it can be concluded that the surface area measurements in the MRI are a repeatable and reliable parameter.

Pritchard et al. verified changes in the volume of the airways in healthy volunteers with the use of various types of inhalers using volumetric MRI (3D) imaging [17]. The maximum values of the glottis area obtained by them were similar to those obtained in our study. On the other hand, different results were obtained by Scheinherr et al., who measured the area of the rima glottidis using a flexible laryngoscope inserted through the nose during both calm and quick breathing [18]. Due to the fact that making objective measurements in laryngoscopy is a significant technical challenge, they applied some assumptions. They allowed for the measurement of the area with the maximum error estimated at 10%. The values obtained by these researchers in the group of healthy men were minimum 1.78 ± 0.3 cm^2^, and maximum 2.17 ± 0.54 cm^2^, while in our measurements, they were as follows: minimum in GRE 0.94 ± 0.3 cm^2^ and in TRUFI 0.68 ± 0.2 cm^2^, maximum for GRE 1.62 ± 0.34 cm^2^ and for TRUFI 1.56 ± 0.38 cm^2^. The data reported by Scheinherr et al. are puzzling because such significant differences should not result solely from the reconstruction method of the laryngoscopic images. Research comparing both measurement methods in terms of their real value would be valuable.

Statistical significance was found for the measurements of maximum areas of the glottis compartments in the TRUFI sequence and the minimum and maximum areas in the GRE sequence for the affected vocal folds. The minimum area for the TRUFI sequence showed significance at the level of statistical trend. The highest statistical significance was characteristic for the maximum area of glottis compartments in the GRE sequence, which proves the highest diagnostic value of this particular technique.

In postoperative patients with vocal fold paralysis, both MRI sequences, TRUFI and GRE, were compared in terms of the maximum values of the angles and the areas of the glottis compartments during phonation. Statistical differences were found for both parameters. The medians for the GRE sequences reached a higher value, and the ranges of angles of vocal folds and areas of the glottis compartments obtained with the two sequences are almost disjoint. A possible explanation for this may be the discrepancies in the thickness and number of layers between the sequences and the insufficient compensation of the effect of changes in larynx position in the TRUFI sequence.

## 5. Conclusions

To sum up, dynamic ultrasound examinations and MRI performed in the 1.5T field are of comparable value to the laryngoscopy in the assessment of vocal fold mobility in patients with goiter, including the detection of postoperative paralysis. The most reliable technique seems to be the GRE sequence in MRI examinations, and the most effective parameter is the maximum area of the glottidis compartment. The possibility of incorporating dynamic short sequences available on 1.5T scanners into standard neck examination protocols is a novelty and a promising, objective diagnostic perspective in the diagnosis of vocal fold paralysis. Undoubtedly, the role of MRI studies in the assessment of the larynx will increase given the current state of high epidemic threat.

## Figures and Tables

**Table 1:** The minimum and maximum angles and the areas of the vocal folds in the group of patients with abnormal postoperative VF mobility obtained during normal breathing via US and MRI.

**Parameter**	**Min. Angle [°]**				**Max. Angle [°]**			
Method	US	MRI-GRE		MRI-TRUFI	US	MRI-GRE		MRI-TRUFI
Female	9.60 ± 2.37 (Me = 10)	5.90 ± 3.21 (Me = 6)		5.38 ± 3.25 (Me = 5.5)	10.6 ± 2.37 (Me = 10.5)	7.50 ± 3.89 (Me = 8)		7.38 ± 2.83 (Me = 6.5)
**Parameter**	**Min. Area [cm^2^]**				**Max. Area [cm^2^]**			
Method	MRI-GRE		MRI-TRUFI		MRI-GRE		MRI-TRUFI	
Female	0.19 ± 0.13 (Me = 0.18)		0.14 ± 0.11 (Me = 0.1)		0.22 ± 0.15 (Me = 0.2)		0.18 ± 0.11 (Me = 0.1)	

**Table 2:** The minimum and maximum angles and the areas of the vocal folds in the group of patients with normal postoperative VF mobility obtained during quiet breathing via US and MRI.

**Parameter**	**Min. Angle [°]**				**Max. Angle [°]**			
Method	US	MRI-GRE		MRI-TRUFI	US	MRI-GRE		MRI-TRUFI
Female US/All MRI	9.70 ± 2.056 (Me = 9)	10.35 ± 2.25 (Me = 10)		10.7 ± 2.39 (Me = 10)	19.15 ± 2.50 (Me = 19.5)	16.85 ± 2.46 (Me = 16)		18.00 ± 2.89 (Me = 17)
**Parameter**	**Min. Area [cm^2^]**				**Max. Area. [cm^2^]**			
Method	MRI-GRE		MRI-TRUFI		MRI-GRE		MRI-TRUFI	
All	0.28 ± 0.09 (Me = 0.25)		0.27 ± 0.07 (Me = 0.25)		0.52 ± 0.14 (Me = 0.50)		0.49 ± 0.11 (Me = 0.50)	

## Data Availability

All data supporting this study will be available after contact with author Magdalena Derlatka-Kochel (magdalena.derlatka@umed.lodz.pl).

## Abbreviations

GRE	generic gradient echo
MRI	magnetic resonance imaging
ROI	region of interest
TRUFI	true fast imaging with steady-state free precession
US	ultrasonography
VF	vocal fold
